# In-office needle arthroscopic assessment after primary ACL repair: short-term results in 15 patients

**DOI:** 10.1186/s40634-022-00528-1

**Published:** 2022-09-07

**Authors:** Alessandro Annibaldi, Edoardo Monaco, Matthew Daggett, Alessandro Carrozzo, Daniele Mazza, Leonardo Previ, Giorgio Rossi, Pierfrancesco Orlandi, Andrea Ferretti

**Affiliations:** 1 AOU Sant’Andrea, La Sapienza University of Rome, Rome, Italy; 2 Kansas City University, Kansas City, MO USA

## Abstract

**Purpose:**

In-office needle arthroscopy has been reported as a diagnostic tool for different knee pathologies. In addition, ACL repair has seen a resurgence with the advent of innovative orthopedic devices. The aim of this study was to assess clinical, radiological, and in-office needle arthroscopic findings in 15 adult patients who underwent acute (within 14 days from injury) anterior cruciate ligament (ACL) repair.

**Methods:**

Fifteen patients voluntarily participated in the study. A second-look arthroscopy was performed with an in-office needle arthroscopy at an average of 7.2 months after the primary repair. The parameters included in the investigation were the continuity of the anatomical footprint of the repaired ACL, subjective assessment of the ACL tension with the probe, and synovial coverage of the ACL. All patients had a Magnetic Resonance Imaging (MRI) at 6 months after repair and an arthrometric evaluation with the KT-1000. Clinical evaluation with the scores, Tegner Lysholm Knee Scoring Scale (TLKSS), the Knee Injury and Osteoarthritis Outcome Score (KOOS), and International Knee Documentation Committee (IKDC) was performed at the final follow-up of 2 years. Moreover, a correlation between the characteristics of ACL appearance at the time of the second look in-office needle arthroscopy, MRI and KT-1000 was performed.

**Results:**

The mean TLKSS was 97.86, the mean KOOS was 98.08 and the mean subjective IKDC was 96.71. The objective IKDC was A in 10 patients and B in 5 patients. ACL healing was graded as A in 11 patients and B in 4 patients. Synovial coverage was graded as good in 10 patients and fair in 5 while MRI assessment showed a type I ACL in 10 patients, type II in 4 patients and type III in 1 patient.

**Conclusion:**

In-office needle arthroscopy is a reliable tool to assess the condition of a repaired ACL. In addition, ACL repair performed in acute proximal tears demonstrated excellent clinical results.

## Introduction

With the advent of modern arthroscopic surgical techniques and new orthopedic devices, there has been renewed interest in primary repair of anterior cruciate ligament (ACL) in certain patient populations with proximal tears [10]. ACL surgery historically consisted of open primary repair in the acute setting [22]. While preliminary results of open ACL repair were encouraging, the long-term results showed higher rates of continued knee instability and pain, thus ACL reconstruction (ACLR) became the gold standard technique for all tear types [8, 12, 13, 15]. ACLR is a reliable technique but is also associated with unique complications such as donor site morbidity and proprioceptive deficit [22]. Although there have been many studies demonstrating good clinical outcomes of the repaired ACL, there is limited evidence investigating the repaired ACL through second-look visualization and arthroscopic probing compared with ACLR [2, 14, 23].

In the last few years, in-office needle arthroscopy has become increasingly popular [5–25]. Gill et al. [10] in a prospective multicenter clinical trial, found that diagnostic in-office arthroscopy had an accuracy, a sensitivity, and a specificity equal to surgical diagnostic arthroscopy. They also assessed an accuracy greater than Magnetic Resonance Imaging (MRI) in intra-articular meniscus tears. Voigt et al. [23] performed a cost analysis between MRI and in-office diagnostic needle arthroscopy for knee and shoulder diagnosis, finding that in-office arthroscopy was more cost-effective, especially in the diagnosis of medial meniscal tears.

The purpose of this study is to report clinical outcomes, in-office second-look needle arthroscopic findings, and MRI findings in a group of 15 volunteers who underwent ACL repair. We hypothesized that in office-arthroscopy could be a reliable diagnostic tool to evaluate healing of a repaired ACL as compared to MRI.

## Methods

### Patient population

Institutional review board was granted for this study (IRB number blinded for journal review). From January 2018 to May 2020, a total of 154 patients with ACL acute tears, within 14 days from injury, were admitted to our institution. Patients were carefully informed pre-operatively about possible surgical procedures depending on the type of lesion and tissue quality of the remnant found intraoperatively. Acute proximal type I and II with good or fair tissue quality, according to the modified Sherman classification by van der List et al. [19], were repaired. Based on these criteria, a total of 49 ACL repair were performed in the study period.

A total of 15 adult patients, who underwent ACL repair, agreed voluntarily to participate in the study. The purpose of second look in-office needle arthroscopy was explained to all patients prior and informed written consent was subsequently obtained.

### In-office arthroscopy technique

A sterile field with the disposable kit was prepared. The kit includes a 20-cc syringe of 1% lidocaine with epinephrine/0.75 ropivacaine, a separate 20-cc syringe with only 0.75% ropivacaine, a saline-filled 60-cc syringe, Chlorhexidine scrub, the needle arthroscopy (NanoScope™ Console, Arthrex, Naples, FL). Patients were placed in the supine position with the knee free to move from full extension to flexion. The leg was draped in a sterile fashion and a stockinette was placed over the foot and ankle and secured in place just distal to the tibial tuberosity with Coban wrap. The standard anteromedial and anterolateral portals were marked 1 cm medial and lateral to patellar tendon. Other working portals were marked medial to the standard medial portal and lateral to the lateral portal. The portals were made by inserting percutaneously the Nanoscope sharp obturator without blade. The 20-mL syringe with a 25- gauge needle was used to infiltrate 10 mL of the mixed local anaesthetic to each portal site and the surrounding capsule to anesthetize the area. Subsequently, an intra-articular injection of 20 cc of 0.75% ropivacaine was performed. The needle arthroscope was connected to the viewing tablet in sterile fashion, and a 60-mL syringe of sterile saline was attached to the inflow port of the needle arthroscopy hand piece (NanoScope™ Handpiece, Arthrex, Naples, FL). The anteromedial portal was used to insert the arthroscopy needle. The arthroscope has a 0° viewing angle and 120° field of view. Saline solution can be injected to the joint with the 60-mL syringe to distend the joint space and remove obstructing tissue blocking the arthroscope. After the insertion of the camera, a standard diagnostic arthroscopy was performed. A NanoProbe (NanoScope™ Probe, Arthrex, Naples, FL) was inserted from one of the accessory portals to evaluate the tension of the repaired ACL. At the end of each procedure an aspiration of the saline solution injected during the procedure was performed before the removal of the device to minimize the patient’s post-operative discomfort. The needle arthroscope was then removed, and the portals were covered with band-aids.

After the in-office arthroscopy procedure, patients were kept for observation for one hour. No patients reported complications and all patients immediately returned to daily activities.

### Patient assessment

**Fig. 1:**
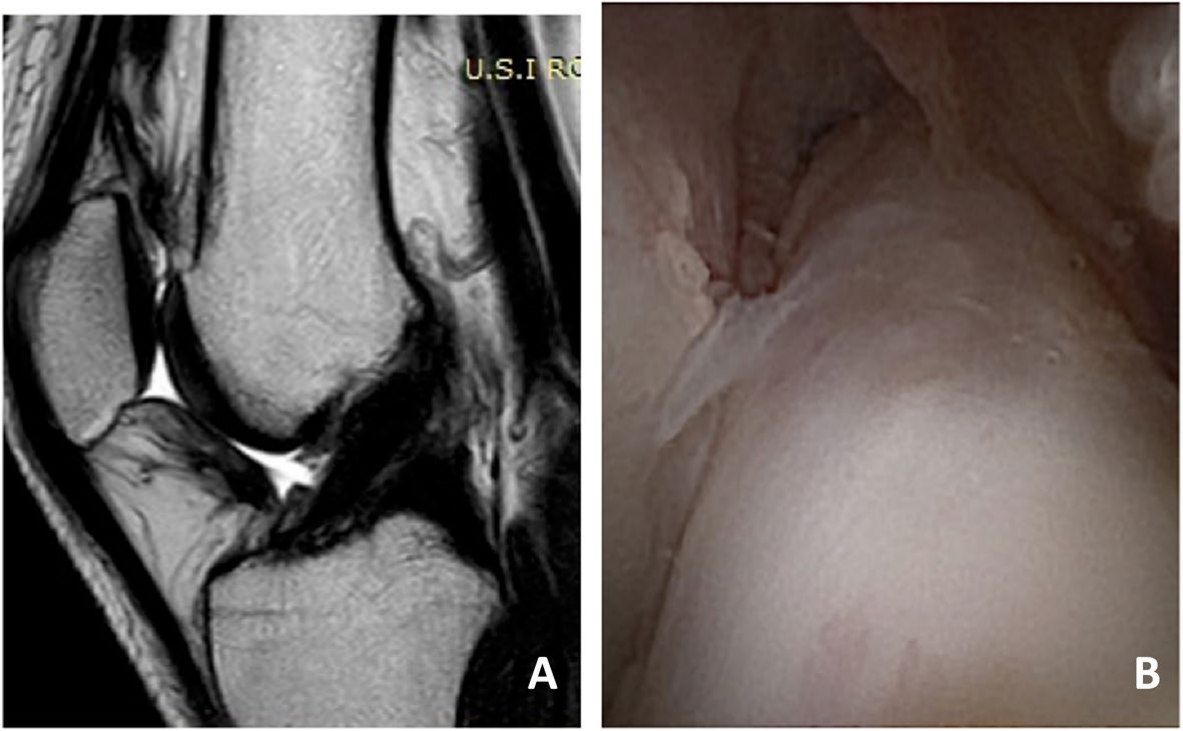
ACL repair. **A** Sagittal T2-TSE MRI post-operative 6 months after ACL repair (Howell I); **B** In-office needle arthroscopic view of the repaired ACL of the same patient showing type A healing

The second look in-office needle arthroscopy was performed at an average follow-up of 7.2 months after ACL repair before allowing patients to return to sports. Moreover, all patients were also evaluated at a mean follow-up of 6 months with 1.5 T MRI and KT-1000 arthrometric measurement (MedDmetric, San Diego, CA) as expected by our standard post-operative protocol. Clinical assessment with patients reported outcomes scores was performed at the final 2-year follow-up using the Tegner Lysholm Knee Scoring Scale (TLKSS), the Knee Injury and Osteoarthritis Outcome Score (KOOS), and International Knee Documentation Committee (IKDC) subjective and objective scores for greater validity. An experienced musculoskeletal radiologist was asked to describe the MRI images and T2- Turbo spin echo (TSE) sequences were considered and ACL maturity was measured with a four-grade system according to Howell et al. [11]: I, homogeneous, low-intensity signal indistinguishable from the PCL and patellar tendon; II, normal ligament signal over at least 50% of its volume, intermingled with portions that have increased signal intensity; III, increased signal intensity over at least 50% of its volume, intermingled with portions that have a normal ligament signal; or IV, diffuse increase in signal intensity without strands with a normal ligament appearance [3] (Fig. 1 A, B).

### In-office needle arthroscopic assessment

**Fig. 2:**
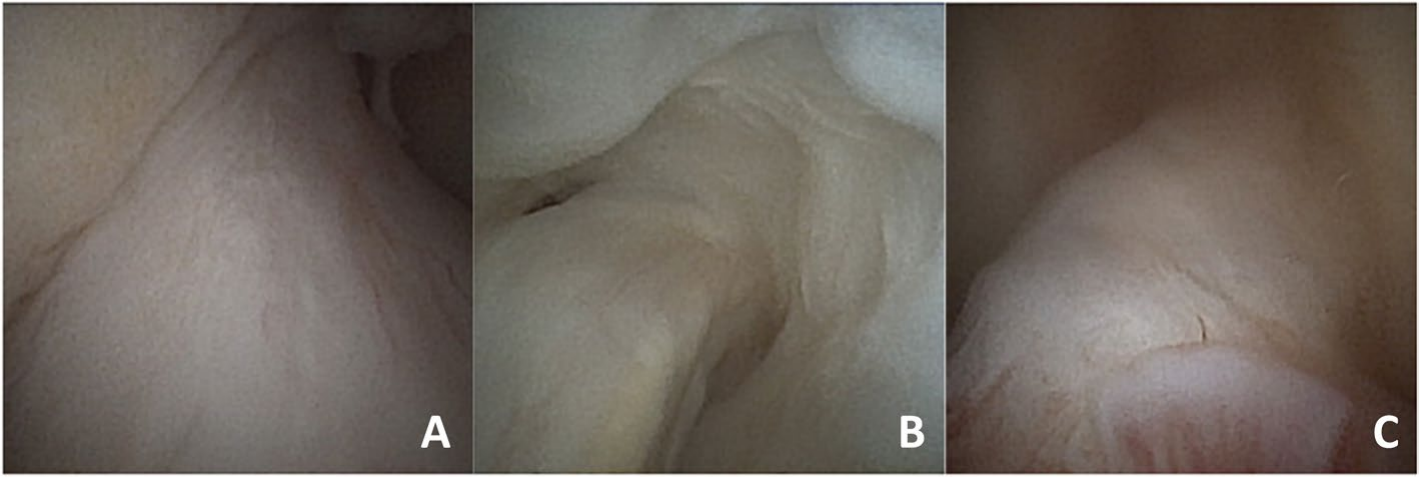
**A**, **B**, **C** Good appearance of synovial coverage of the repaired ACL with in-office needle arthroscopy

**Fig. 3:**
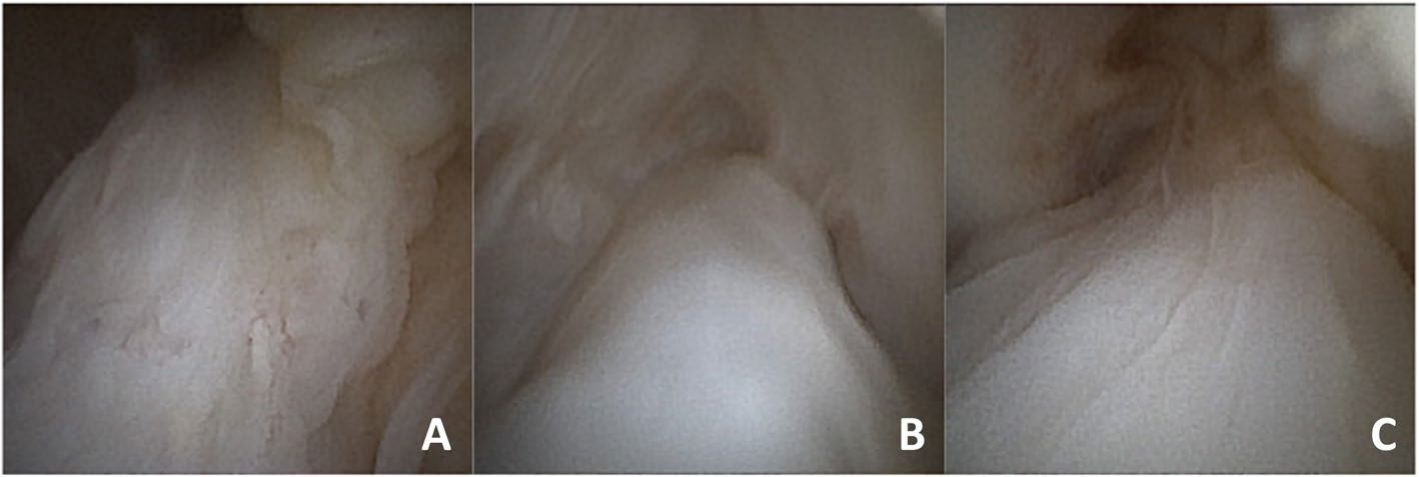
**A**, **B**, **C** Fair appearance of synovial coverage of the repaired ACL with in-office needle arthroscopy

The in-office needle arthroscopic second look focused on the evaluation of the continuity and anatomic footprint (healing) of the repaired ACL, subjective assessment of the ACL tension with the probe and evaluation of the synovial coverage. The healing of repaired ACL was graded by the examiner in four subjective types. Type A is a normal adhesion to the femoral footprint with excellent anatomical continuity (Fig. 1B); type B is a nearly normal adhesion to the femoral footprint with good anatomical continuity; type C is a moderate adhesion to the femoral footprint and fair anatomical continuity and D is abnormal adhesion to the femoral footprint with poor anatomical continuity. Tension has also been classified into four subjective types: normal tension (A), slight laxity (B), fair laxity (C) and poor laxity (D). Synovial coverage of the repaired ligament was classified into 3 categories: good, when the synovium entirely covered the repaired ligament (Fig. 2); fair, when the area of synovial coverage was more than 50% of the entire area (Fig. 3); poor, when more than 50% of the repaired ligament was without synovial coverage. A comparison between the appearance of the repaired ACL with second look was performed in relation to the MRI appearance of the ACL (signal intensity of the repaired ACL based on the Howell scale), the subjective tension based on ligament probing and arthrometric evaluation with KT-1000.

### Statistical analysis

All analyses were performed with SPSS for MacOS (v 27.0.1.0; IBM). Descriptive data analysis was conducted depending on the nature of the considered criteria. The mean, range, frequencies and proportions of demographic data and clinical outcomes were calculated. The correlation between the characteristics of ACL appearance at the time of the second look arthroscopy was investigated with the Kendall tau rank correlation coefficient.

## Results

During the study period a total of 15 volunteers agreed to participate in the study. The demographics of each patient, clinical outcomes, in-office arthroscopic evaluation of the repaired ACL, the side-to-side difference at KT-1000, the synovial coverage and the Howell grade at MRI are presented in Table 1. There were 11 males and 4 females. The mean age was 33.1 years (range 21–55 years), and the mean follow-up was 7.2 months (range 6–10 months). The mean duration of the second look in office arthroscopic procedure was 12 min (range 8–15 min). The mean TLKSS was 97.5, the mean KOOS was 98.1 and the mean subjective IKDC was 96.7. The objective IKDC was A in 10 patients and B in 5 patients. KT-1000 measurements showed a maximum manual side-to-side difference of less than 2 mm in ten patients, whereas five patients showed a difference of 3 mm. ACL healing was graded A in 11 patients and B in 4 patients, synovial coverage was graded good in 10 patients and fair in 5 patients while MRI assessment according to Howell scale showed a type I ACL in 10 patients, type II in 4 patients and type III in 1 patient.

**Table 1:** Patient demographic data, clinical outcomes at 2 years follow-up, and in-office needle arthroscopic findings of the study population

	Age	Sex	Side	In-office needle arthroscopy mean FU (Months)	Procedure Duration (minutes)	TLKSS	KOOS	Other symptoms	Pain	ADL	Sport/rec	QOL	Subjective IKDC	Objective IKDC	Difference on KT-1000 maximum manual testing at 30° (mm)	ACL healing	ACL tension	Synovial Coverage	MRI Howell Grade
Patient 1	28	M	Left	8	8	100	100	100	100	100	100	100	100	A	0	A	A	Good	I
Patient 2	30	F	Right	10	15	95	97.6	96.4	100	98.5	95	93.7	97.7	B	3	B	B	Fair	II
Patient 3	24	F	Right	6	12	100	100	100	100	100	100	100	98.9	A	2	A	A	Good	I
Patient 4	21	M	Right	7	11	100	99.4	96.4	100	100	100	100	95.4	B	3	A	A	Fair	II
Patient 5	38	M	Left	8	13	95	98.8	96.4	100	100	95	100	97.7	A	1	B	B	Good	III
Patient 6	34	M	Right	6	14	100	99.4	100	97.2	100	100	100	100	A	1	A	A	Good	II
Patient 7	23	M	Right	7	8	95	95.8	96.4	97.2	97.1	90	93.7	93.1	B	3	A	A	Fair	I
Patient 8	43	F	Left	7	12	98	98.8	100	97.2	100	95	100	95.4	B	3	B	B	Good	I
Patient 9	55	M	Right	8	12	98	97.6	96.4	100	97.1	95	100	98.9	A	2	A	A	Good	I
Patient 10	22	M	Left	7	15	91	94.6	92.9	97.2	95.6	90	93.7	95.4	A	2	A	A	Fair	I
Patient 11	42	M	Right	6	11	98	97.6	96.4	97.2	98.5	95	100	93.1	A	1	A	A	Good	I
Patient 12	40	M	Right	7	13	100	100	100	100	100	100	100	100	A	0	A	A	Good	I
Patient 13	52	F	Right	6	10	95	94	96.4	97.2	94.1	90	93.7	89.7	B	3	B	B	Fair	II
Patient 14	23	M	Right	8	12	98	97.6	96.4	97.2	98.5	95	100	95.4	A	2	A	A	Good	I
Patient 15	22	M	Right	7	14	100	100	100	100	100	100	100	100	A	1	A	A	Good	I
Mean ± SD	33.1 ± 11.3			7.2 ± 1.1	12 ± 2.2	97.5 ± 2.7	98.1 ± 2	97.6 ± 2.2	98.7 ± 1.4	98.6 ± 1.9	96 ± 3.9	98.3 ± 2.9	96.7 ± 3.1		1.8 ± 1.1				

*FU* Follow-up, *TLKSS* Tegner Lysholm Knee Scoring Scale, *KOOS* Knee Injury and Osteoarthritis Outcome Score, *ADL* Activities of daily living, *QOL* Quality of life, *IKDC* International Knee Documentation Committee, *ACL* Anterior cruciate ligament, *MRI* Magnetic Resonance Imaging, *SD* Standard deviation

With the previously described criteria, ACL healing features positively and significantly correlated with ACL tension (b = 0.7, *P* = 0.006), Howell grade (b = 0.575, *P* = 0.021) and with the side-to-side difference measured at KT-1000 (b = 0.596, *P* = 0.013). Still, ACL tension showed a positive significant correlation with side-to-side difference in anterior tibial translation (b = 0.533, *P* = 0.026). Details regarding the correlation between variables are displayed in Table 2.

**Table 2:** Evaluated criteria and their correlation

	**ACL Healing**	**ACL Tension**	**Howell grade**	**KT—1000**
**ACL Healing**				
Correlation coefficient	n.a	.704	.575	.596
Significance	n.a	.006	.021	.013
**ACL Tension**				
Correlation coefficient	.704	n.a	.3	.533
Significance	.006	n.a	.225	.026
**Howell grade**				
Correlation coefficient	.575	.3	n.a	.276
Significance	.021	.225	n.a	.237
**KT—1000**				
Correlation coefficient	.596	.553	.276	n.a
Significance	.013	.026	.237	n.a

*ACL* Anterior cruciate ligament tears, *n.a.* Not applicable

## Discussion

The main finding of the current study is that in-office needle arthroscopy is a reliable and safe diagnostic tool that allows the ability to assess the healing, the tension, and the synovial coverage of a repaired ACL through direct visualization and probing. Moreover, a statistically significant correlation was found between ACL healing, tension and MRI appearance according to Howell scale and side-to-side difference as evaluated with KT-1000. A correlation between the MRI signal and the biomechanical properties of the anterior cruciate ligament has already been shown by Weiler et al. [24] in a study performed at 2-year follow-up to evaluate the fate of an ACL graft in sheep. They found that high signal intensity on MRI revealed a reduction in the mechanical properties of the graft during the first stages of remodelling. Our study also demonstrated that ACL repair in this patient population can be considered a viable alternative to ACLR.

Accurate patient selection is essential to achieve good clinical outcomes as assessed by Sherman et al. [17] in 1991 and confirmed by numerous subsequent studies [1, 19]. The renewed interest in ACL repair encouraged several authors to show their clinical and radiological outcomes. Di Felice et al. [7] showed excellent results at a mean 3.5- year follow-up after ACL repair with a failure rate of 9% while Achtnich et al. [1], who compared primary ACL repair with ACLR, pointed out similar outcomes with a trend toward more revision after primary repair. In a recent study, Ferretti et al. [9] evaluated in a consecutive series of acute proximal ACL tears, morphology (normal or abnormal) and signal intensity graded against posterior cruciate ligament signal intensity in isointense, intermediate and hyperintense. They found good MRI findings with a normal morphology and signal intensity after ACL repair. Over the past few years, several techniques have been proposed for ACL repair. A recent meta-analysis that evaluated 1101 patients treated with different ACL repair techniques showed a failure rate between 7 and 11% with good outcomes at a mean follow-up of 2.1 years [20]. Another recent systematic review in which the differences between primary ACL repair and ACLR regarding Lysholm score, IKDC, side-to-side laxity difference, pivot shift test, and graft rupture were analyzed showed no significant differences between the two techniques. In addition, excellent mid- and long-term results were shown with the suture anchor repair technique [13].

The in-office needle arthroscopy technology has mainly been reported as a diagnostic tool and has also recently been described as a tool in the treatment of knee, shoulder, and ankle pathologies [5, 16]. Diagnostic in-office shoulder needle arthroscopy allows for a diagnosis at the first patient encounter, especially in pathologies where MRI has low sensitivity such as partial rotator cuff tears. Other recent studies showed a greater diagnostic accuracy using in-office needle arthroscopy as compared with MRI for meniscal tears, chondral defect, and other non-ligamentous pathology [25]. Colasanti et al. [4] found several benefits of using in-office needle arthroscopy for anterior ankle impingement such as quicker patient recovery, patient satisfaction, and cost reduction.

Cost reduction is one of the most repeated themes in several papers on needle arthroscopy. In fact, the cost reduction compared with MRI, already highlighted by Voigt et al. [23] for meniscal injuries, has also been subsequently confirmed by McMillan et al. [16]. They retrospectively reviewed 200 patients undergoing in-office needle arthroscopy diagnostic procedure (175 knees and 25 shoulders) and compared the reimbursements for in-office needle arthroscopy with the cost of diagnostic MRI, finding savings for patients in both the knee and shoulder.

In-office needle arthroscopy was also used to assess the status of a surgically repaired meniscus. DiBartola et al. [6] demonstrated through in-office needle arthroscopy the healing of four repaired horizontal cleavage tears of the medial meniscus and four of the lateral meniscus six months after surgery.

To our knowledge, there is no study on the results of second-look needle arthroscopy of repaired anterior cruciate ligament. There are, however, several second-look arthroscopic reports on reconstructed ACL. Ahn et al. [2] in a previous study performed on 208 patients undergoing anterior cruciate ligament reconstruction with patella bone-tendon-bone (PBTB) autograft or hamstring tendon at a mean follow-up of 21 months, evaluated graft continuity, subjective tension with the probe, and subjective synovial coverage. They demonstrated that the hamstring graft had better synovial coverage than the bone-patellar tendon-bone graft. In contrast, a more recent study by Mae et al. [14], which evaluated reconstructed ACL tension, graft damage, synovial coverage on 113 patients at a mean follow-up of 10 months demonstrated that the bone-patellar tendon- bone was better than the hamstring in anatomic ACLR.

In our study, the clinical outcomes of ACL repair at 2 years of follow-up showed excellent scores and a resumption of normal daily activities and sports in all 15 patients examined. The macroscopical appearance of the ligament was excellent to very good in all cases as well as the subjective tension and synovial coverage. In addition, in-office needle arthroscopy was well tolerated, without complication and allowed immediate return to daily activities.

This study has some limitations: the first is the relatively small sample size and the short follow-up period between the primary ACL repair and the second arthroscopic examination. Another limitation of the study is the lack of a control group of ACLR and related MRI.

## Conclusion

In-office needle arthroscopy is a reliable tool to assess the condition of a repaired ACL. In addition, ACL repair performed in acute proximal tears demonstrated excellent clinical results.

## Data Availability

Data are available from the corresponding author on reasonable request.

## Abbreviations

ACL: Anterior cruciate ligament
ACLR: Anterior cruciate ligament reconstruction
MRI: Magnetic resonance imaging
TLKSS: Tegner Lysholm Knee Scoring Scale
KOOS: Knee Injury and Osteoarthritis Outcome Score
ADL: Activities of daily living
QOL: Quality of life
IKDC: International Knee Documentation Committee
SD: Standard deviation
